# Supplementary material for: MetaQTL: a package of new computational methods for the meta-analysis of QTL mapping experiments
Source: BMC Bioinformatics. 2007 Feb 8;8:49. doi: 10.1186/1471-2105-8-49 (PMC1808479; doi:10.1186/1471-2105-8-49)
Supplement: Additional File 7 — MetaQTL Package : jar file and tutorial. This Zip archive contains both the MetaQTL JAR file and the files of the tutorial. [file 1471-2105-8-49-S7.zip › org.inra.metaqtl/doc/org/metaqtl/graph/Layer.html]

Layer


|  |  |  |
| --- | --- | --- |
| **Overview** \| **Package** \| **Class** \| **Use** \| **Tree** \| **Deprecated** \| **Index** \| **Help** | |  |
| **PREV CLASS**   **NEXT CLASS** | **FRAMES**    **NO FRAMES**     **All Classes** |  |
| SUMMARY: NESTED \| FIELD \| CONSTR \| METHOD | DETAIL: FIELD \| CONSTR \| METHOD |  |


---


## org.metaqtl.graph Class Layer

```
java.lang.Object
  org.metaqtl.graph.AnchoredElement
      org.metaqtl.graph.Layer
```

**Direct Known Subclasses:**: ChromLayer, QtlLayer, QtlPartitionLayer, QtlTreeLayer

---

``` public abstract class Layer extends AnchoredElement ```

---

| **Field Summary** | |
| --- | --- |
| `protected  double` | `height`             The height of the layer. |
| `protected  double` | `width`             The width of the layer. |

**Fields inherited from class org.metaqtl.graph.AnchoredElement**

`x, y`


**Constructor Summary**

`Layer(double x,
double y)`
  
          Creates a layer without any width and heigth
constraints which upper left corner will be
located at the point (x,y).


| **Method Summary** | |
| --- | --- |
| `abstract  void` | `attach(java.lang.Object object)`             Attach the given `object` to the layer. |
| `abstract  void` | `build(java.awt.Graphics2D graph)`             Build the layer. |
| `abstract  void` | `draw(java.awt.Graphics2D graph)`             Draws the layer on the given `graph` context. |
| `double` | `getHeight()` |
| `double` | `getWidth()` |
| `abstract  double` | `getYMax()` |
| `abstract  double` | `getYMin()` |

**Methods inherited from class org.metaqtl.graph.AnchoredElement**

`getX, getY, setX, setY`

**Methods inherited from class java.lang.Object**

`clone, equals, finalize, getClass, hashCode, notify, notifyAll, toString, wait, wait, wait`

**Field Detail**

### width

```
protected double width
```

:   The width of the layer.

---


### height

```
protected double height
```

:   The height of the layer.


**Constructor Detail**

### Layer

```
public Layer(double x,
             double y)
```

:   Creates a layer without any width and heigth
    constraints which upper left corner will be
    located at the point (x,y).

    **Parameters:**: `x` - the abscisse of the upper left corner of the layer.: `y` - the ordinate of the upper left corner of the layer.


**Method Detail**

### attach

```
public abstract void attach(java.lang.Object object)
```

:   Attach the given `object` to the layer.

    :   **Parameters:**: `object` - the object to attach to the layer.

---


### build

```
public abstract void build(java.awt.Graphics2D graph)
```

:   Build the layer.

---


### draw

```
public abstract void draw(java.awt.Graphics2D graph)
```

:   Draws the layer on the given `graph` context.

    :   **Parameters:**: `graph` -

---


### getHeight

```
public double getHeight()
```

:   **Returns:**: Returns the height.

---


### getWidth

```
public double getWidth()
```

:   **Returns:**: Returns the width.

---


### getYMin

```
public abstract double getYMin()
```

---


### getYMax

```
public abstract double getYMax()
```


---


|  |  |  |
| --- | --- | --- |
| **Overview** \| **Package** \| **Class** \| **Use** \| **Tree** \| **Deprecated** \| **Index** \| **Help** | |  |
| **PREV CLASS**   **NEXT CLASS** | **FRAMES**    **NO FRAMES**     **All Classes** |  |
| SUMMARY: NESTED \| FIELD \| CONSTR \| METHOD | DETAIL: FIELD \| CONSTR \| METHOD |  |


---
